# A qualitative study exploring and understanding the experiences and unmet needs of adults with type 2 diabetes and diabetes‐related foot ulcers

**DOI:** 10.1111/dme.70343

**Published:** 2026-05-04

**Authors:** Molly Caba, Jane Speight, Agnieszka Glab, Patrick Highton, Kamlesh Khunti, Michelle Hadjiconstantinou

**Affiliations:** ^1^ Diabetes Research Centre, College of Life Sciences University of Leicester Leicester UK; ^2^ School of Psychology, Institute for Health Transformation Deakin University Geelong Victoria Australia; ^3^ The Australian Centre for Behavioural Research in Diabetes (ACBRD), Diabetes Victoria Carlton Victoria Australia; ^4^ Leicester Diabetes Centre University Hospitals of Leicester, NHS Trust Leicester UK; ^5^ NIHR Applied Research Collaboration East Midlands Leicester UK; ^6^ NIHR Leicester Biomedical Research Centre Leicester UK

**Keywords:** cardiovascular disease, diabetic foot ulcer, qualitative, type 2 diabetes mellitus

## Abstract

**Aims:**

Diabetes‐related foot ulcers (DFUs) are associated with depression, impaired health‐related quality of life, an increased risk of cardiovascular disease and early mortality. To inform holistic care pathways, this qualitative study explored the experiences and unmet needs of adults living with type 2 diabetes mellitus (T2DM) and DFUs.

**Methods:**

Semi‐structured interviews were conducted with 25 adults with T2DM and current or previous DFUs, and 20 healthcare professionals with experience treating DFUs. Topic guides were underpinned by the Theoretical Domains Framework. Data were analysed using reflexive thematic analysis.

**Results:**

Four themes and three sub‐themes were created relating to the impact of DFUs. DFUs were perceived as a ‘wake‐up call’; the experience often came as a shock, prompting individuals to consider what was responsible for their development. DFUs impacted individuals' behaviours in multiple ways: for some, they were a catalyst for positive change; for others, they fostered behaviours that were unhealthful and/or discordant with professionals' advice. DFUs negatively affected the physical and psychosocial well‐being of individuals with DFU; sedentary behaviours instigated by DFUs led to changes in mood and mental health, in addition to increased weight, mobility problems and an amplified risk of health complications. Regarding the unmet needs of people with T2DM and DFUs, three themes were developed, capturing the need for treatment plans created through shared decision making and improving access to physical well‐being and psychological support.

**Conclusions:**

These findings identify several important areas of unmet need regarding care for adults with T2DM and DFUs, which can help inform improved support for this population.

**What's new?**

- Among those with type 2 diabetes mellitus (T2DM), diabetes‐related foot ulcers (DFUs) are associated with psychological and physical ill‐health, including an amplified risk of depression and cardiovascular disease. Holistic care pathways are needed to better meet the needs of this high‐risk population.
- Individuals with T2DM are largely unaware of DFUs prior to their development. A diagnosis is associated with positive and negative changes in health behaviours, alongside adverse changes in psychosocial and physical health which receive limited support.
- Improved DFU education is needed, alongside tailored behaviour change support and improved access to psychological and physical health interventions encompassing non‐weight‐bearing activities.

## 1 INTRODUCTION

Diabetes‐related foot ulcers (DFUs) are open lesions below the ankle that are a severe complication of diabetes, often preceded by neuropathy (nerve damage) and ischaemia (reduced blood flow).^1^, ^2^ Globally, the prevalence of DFUs among individuals with type 2 diabetes mellitus (T2DM) is 48.9%.^3^ For adults with DFUs, cardiovascular disease (CVD) is the leading cause of death, with their development indicating extensive vascular damage and inflammation.^4^ DFUs are further associated with impaired health‐related quality of life,^5^ increased anxiety^6^ and premature mortality.^7^ Nearly 50% of individuals with DFU report depressive symptoms,^8^ in comparison to 19% of individuals with T2DM without DFUs.^9^

Impaired psychological well‐being has been linked to reduced cardiovascular health through biological, behavioural and psychosocial pathways.^10^ For example, through increased platelet build‐up and inflammation (high levels of stress increasing cortisol levels), engagement in harmful health behaviours (e.g. physical inactivity, unhealthful dietary behaviours) and lower levels of social support decreasing one's ability to manage life stressors. As such, the already increased CVD risk experienced by individuals with DFU may be further exacerbated by their reportedly low psychological well‐being.

In contrast, positive psychological well‐being (e.g. optimism)^10^ is associated with cardiovascular benefits regardless of physical health status.^11^ This is through a positive association with healthful behaviours (e.g. physical activity) and a negative association with unhealthful behaviours.

Two qualitative meta‐syntheses exploring the experiences of individuals with lived experience of DFUs^12^, ^13^ highlight a number of barriers to engagement with healthcare and healthy habits. Key findings indicate this group often report being unaware of the condition prior to its development, perceive a lack of personal control over its trajectory, and feel unsupported by healthcare professionals (HCPs) both emotionally and clinically, indicating their healthcare needs are not being met. This is of crucial importance given the amplified risk of early mortality through CVD, and impaired psychological health of the T2DM and DFU population.

Therefore, the aim of this study was to elucidate the experiences and current unmet needs of people with T2DM and DFUs to help inform holistic care pathways that best support the population's physical and psychological health. We interviewed both people living with DFU and HCPs, as to help inform care pathways, it is important to understand the experiences and perspectives of those who are the recipients of DFU care and those who are delivering DFU care.

## 2 METHODS

### 2.1 Design

This multi‐centre (University Hospitals of Leicester and University Hospitals of Derby and Burton) qualitative interview‐based study received ethical approval from the Brent Research Ethics Committee, London (22/PR/0272). This was part of a wider NIHR‐funded programme grant, the MiFoot project (NIHR 202021), which aimed to prevent CVD events in people with T2DM and DFU. The Reflexive Thematic Analysis Reporting Guidelines (RTARG) guided reporting of the study.^14^

### 2.2 Participants

Eligible participants were: (a) adults (aged ≥18 years) living with T2DM and current/previous DFUs (diagnosed with DFU ≤3 years previously) or (b) HCPs with experience treating DFUs. As this qualitative study was part of a wider programme grant, our eligibility criteria aligned with the eligibility criteria of the MiFoot project at the time of this study.^15^

### 2.3 Recruitment

People with T2DM and current/previous DFUs, and HCPs were recruited from Leicester and Derby primary and secondary care (see Figure 1 for recruitment pathways for people with T2DM/DFUs, and Figure 2 for HCPs). Those who expressed interest were contacted via telephone or email by M.C. to arrange an interview. A convenience and snowball (HCPs only) approach was adopted. A purposive sampling technique based on variation in gender, education, ethnicity and profession was anticipated. However, challenges recruiting within the study timeframe resulted in the use of convenience sampling.

**FIGURE 1:**
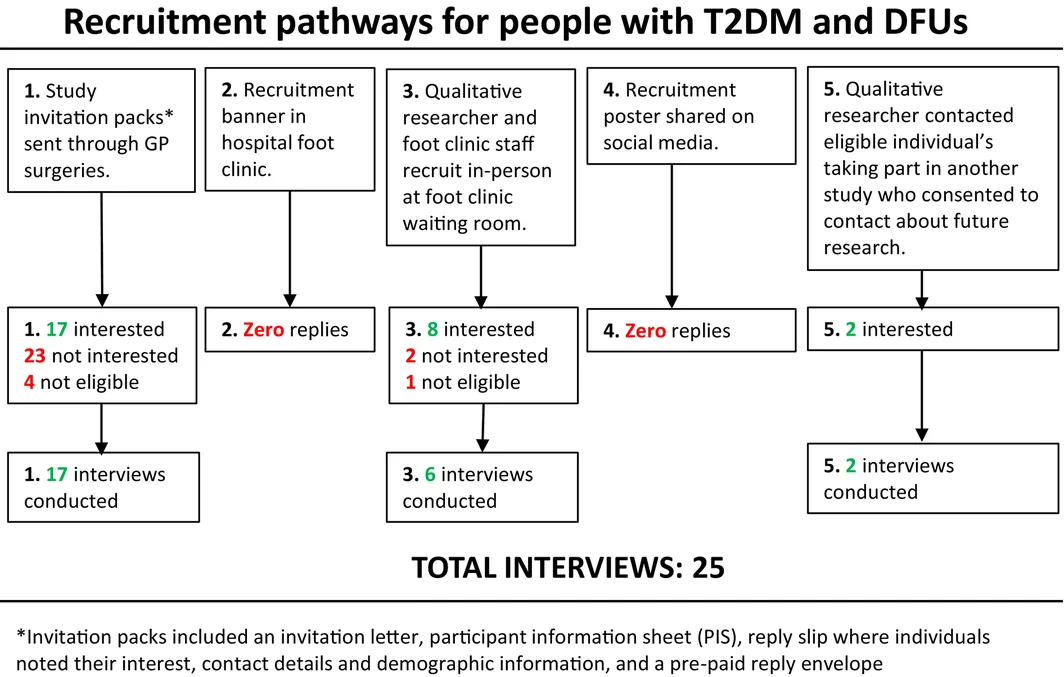
Recruitment pathways for people with T2DM and DFUs.

**FIGURE 2:**
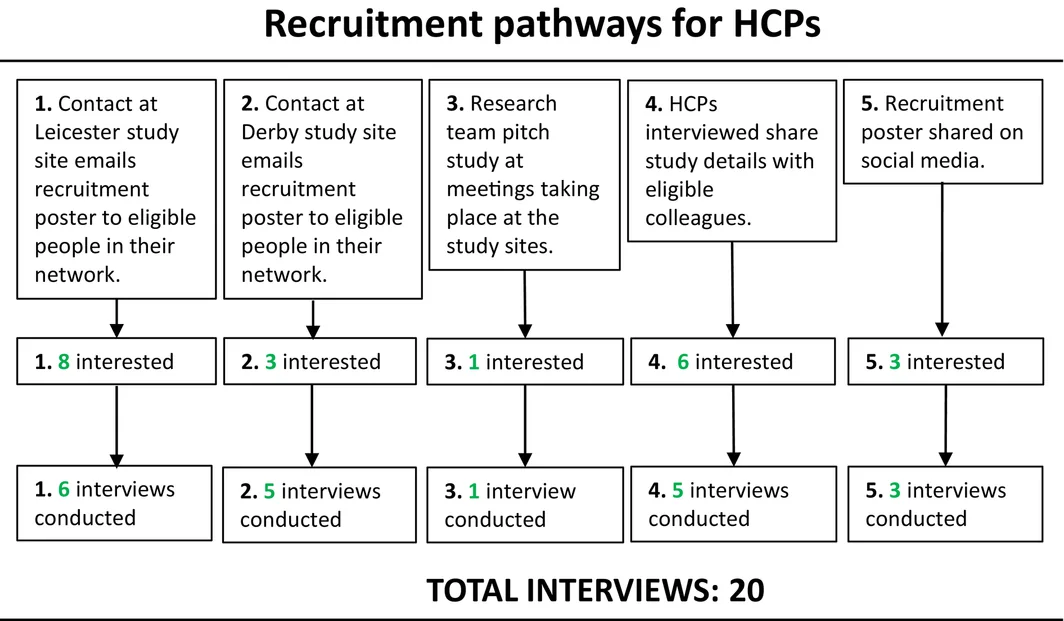
Recruitment pathways for HCPs.

### 2.4 Data collection

Interviews took place either in‐person, via telephone or Microsoft Teams, between August 2022 and January 2023. Initial interviews were co‐facilitated by MC and MH. Co‐facilitation, alongside field notes, were used to ensure data collection aligned with the research aim and to assist with post‐interview reflections. MC conducted the remaining interviews. Prior to the interviews commencing, informed consent was obtained. Audio recordings of the interviews were transcribed verbatim by an independent transcription company. Anonymised transcripts were uploaded to QSR NVivo 12 for data analysis. Interviews with people with T2DM and DFUs averaged 50 min (shortest = 25; longest = 82). Interviews with HCPs averaged 47 min (shortest = 29; longest = 58).

Separate semi‐structured topic guides (see Supporting Information) were created by MC and MH for each set of participants: people with T2DM/DFUs and HCPs. Questions included were informed by the Theoretical Domains Framework (TDF),^16^ which is a framework commonly used to identify factors influencing health behaviours. Factors explored included knowledge, emotion, skills and social influences.

### 2.5 Researcher positionality

MC is a White female researcher experienced in conducting behavioural science‐based qualitative research across different populations living with and helping manage diabetes, including type 1 diabetes, gestational diabetes and T2DM. This was her first experience interviewing individuals with DFU. MH is a senior female qualitative researcher and a behavioural scientist with extensive experience in diabetes management. MH supported MC by meeting throughout the data collection, data coding and data interpretation processes. MC directed the interpretation and synthesis of the findings, supported by meetings with MH to elicit reflexivity. Both researchers had no prior relationship with any of the participants interviewed within this study.

### 2.6 Data analysis

Inductive data analysis was implemented following reflexive thematic analysis principles.^17^ Whilst the TDF was used to inform the topic guides, it was not used to deductively analyse the data. An inductive approach was taken to facilitate a flexible, interpretive approach to developing themes. The theoretical perspective underpinning the analysis was constructivism, whereby each person's reality is informed by their individual perceptions and lived experiences, meaning no one reality is shared. Semantic and latent coding were used to interpret the immediate and underlying meaning of each participant's experiences of T2DM and DFUs. Transcripts were read and re‐read by MC to enable full familiarisation with the data. Semantic codes were then created, with regular meetings taking place between MC and MH to implement latent level coding, and to critically discuss and refine the coding process together with any unrealised biases. Early themes were constructed and reviewed during subsequent meetings with MH, and later critically discussed with a third co‐author (JS). Themes were developed using both sets of participant data to create a shared narrative which identifies and delineates similarities and differences across both datasets. Following this, themes were refined, and data extracts relating to a theme were re‐read to ensure relevance. Transcripts were then re‐read to code any data potentially missed during initial coding. Themes were further reviewed to create sub‐themes, and reflective theme titles were agreed upon.

## 3 RESULTS

In total, 25 interviews were conducted with people with T2DM (see Table 1). The ethnicity of all participants was White (100%). Most were men (84%), aged 60–75 years (64%), and had been diagnosed with T2DM for >10 years (68%), with a current DFU (72%). Twenty interviews were conducted with HCPs (see Table 2). Most were women (85%), podiatrists (75%) and had over nine years' DFU experience (65%).

**TABLE 1:** Sociodemographic characteristics of people with T2DM and DFUs (*n* = 25).

Characteristic	*n*	%
Gender		
Men	21	84
Women	4	16
Age (years)		
40–59	3	12
60–75	16	64
75+	6	24
Ethnic group		
White	25	100
Highest level of education		
Primary school	1	4
Grammar/secondary/high school	8	32
Technical college/diploma	2	8
Apprenticeships/vocational training	8	32
Undergraduate degree	5	20
Postgraduate degree	1	4
Time diagnosed with T2DM (years)		
1–3	1	4
3–10	7	28
10+	17	68
Current or previous DFU		
Current	18	72
Previous	7	28

**TABLE 2:** Sociodemographic characteristics of HCPs (*n* = 20).

Characteristic	*n*	%
Gender		
Men	2	15
Women	18	85
Profession		
Doctor	2	10
Nurse	1	5
Podiatrist	15	75
Healthcare assistant	1	5
Trainee nurse associate	1	5
Years of experience in profession		
1–3	2	10
9+	18	90
Years of experience treating DFUs		
1–3	3	15
5–7	1	5
7–9	3	15
9+	13	65
Qualifications held		
GCSE only	2	10
Undergraduate degree	18	90
Postgraduate degree	10	50
Postgraduate diploma	3	15
Postgraduate certificate	6	30
PhD	2	10
Member of the Royal College of Physicians	1	5
Advanced Clinical Practice	1	5

In relation to the impact of DFUs, four themes and three subthemes were developed: (1) DFUs were perceived as a wake‐up call, (2) Behavioural impact of DFUs, (2i) Catalyst for positive change, (2ii) Fostering unhealthful behaviours, (2iii) Behaviours discordant with treatment advice, (3) Physical impact of DFUs and (4) Psychosocial impact of DFUs. Regarding the unmet needs of people with T2DM and DFUs, three themes were developed: (1) Shared decision making to aid the development of appropriate treatment plans, (2) Psychological support and (3) Physical well‐being support. See Figure 3 for themes and sub‐themes.

**FIGURE 3:**
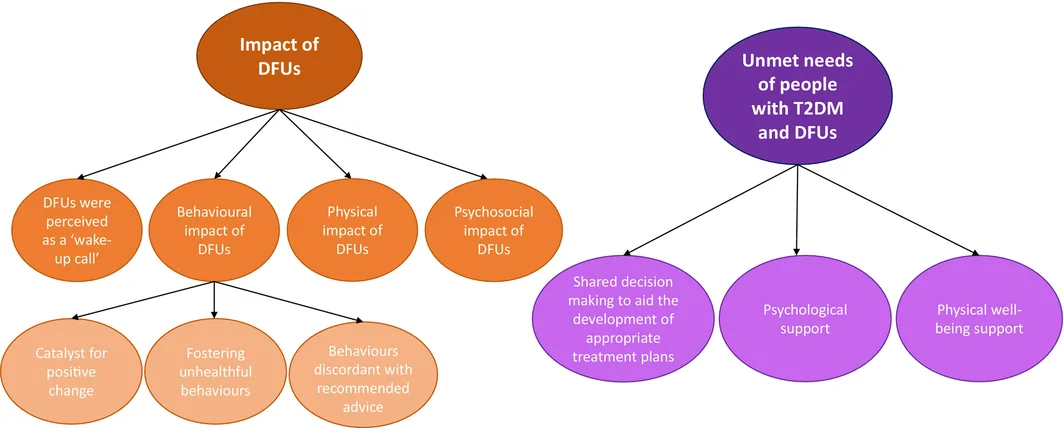
Themes and sub‐themes describing the impact of DFUs and unmet needs of people with T2DM and DFUs.

### 3.1 Impact of DFUs

This theme captures the different ways in which the condition affected individuals' perceptions of T2DM and DFUs as health conditions, their behaviours and their physical and psychosocial well‐being.

#### 3.1.1 DFUs were perceived as a ‘wake‐up call’

Prior to developing a DFU, individuals with T2DM were often unaware foot ulcers were a complication of diabetes, resulting in a surprise or shock reaction to a DFU diagnosis.

People who come into the foot clinic for the first time … are often really surprised that diabetes affects their feet. (HCP8_Podiatrist_9+ years' DFU experience)

Experiencing DFUs and associated complications forced individuals to realise how serious T2DM can be, and the significant impact it can have. For some, the wake‐up call came from their personal DFU experience; for others, it came from witnessing others with DFU complications.

When you go into the diabetic foot clinic … and you see people with lumps missing … feet, lower legs, complete legs. It makes you realise how serious it is, more than any other way. (PWD21_Male_60–75 years)

People who go to the foot clinic for the first time and they see people sat there with amputations or special boots … suddenly get a wakeup call. (HCP21_Podiatrist_9+ years' experience)

Experiencing DFUs prompted individuals with T2DM to consider potential reasons for their development, and where responsibility lay. Some blamed themselves, discussing delayed help‐seeking behaviours, being careless towards their neuropathy and having a ‘blasé’ attitude towards T2DM. Many expressed regrets for not taking their T2DM diagnosis seriously enough, not actively managing their condition, and identifying particular behaviours they believed caused their DFU:

I've had a rather flippant attitude to diabetes … over the years I've thought I could eat and drink what I want and not have any consequences. Well, I've learned from my cost of losing a leg that I was wrong and I should have paid more attention to the warnings I was getting. (PWD23_Male_60–75 years)

However, individuals with T2DM did not feel wholly responsible for their DFU development, with many indicating that they were not adequately informed by HCPs about the condition. As a result, they wanted improved T2DM and DFU education, and wondered whether appropriate education early on or at all, would have avoided DFUs and associated complications:

I do wonder if, with better education a lot earlier on, we could have avoided all this. We don't know … every individual is different. But it's a question I do ask myself. (PWD9_Male_60–75 years)

In contrast, HCPs reported that people with T2DM are invariably told about DFUs, but many factors can affect understanding, or retention of such information. For example, low health literacy, use of complicated jargon and the HCP perception that individuals may be *‘in denial’*:

Just giving information leaflets may not be helpful because actually they either may not have time to look at it and there may be a literacy barrier. (HCP3_Doctor_5–7 years' experience)

#### 3.1.2 Behavioural impact of DFUs

DFUs impacted people's behaviours in different ways, both positively and negatively. For some, they acted as a catalyst for positive change, whereas for others, they fostered unhealthy behaviours and led to behaviours that were discordant with treatment advice.

##### 3.1.2.1 Catalyst for positive change

For some, DFUs acted as a catalyst for positive change. Individuals sought to improve their health and avoid ulcer recurrence through behaviours they believed would help. This included reduced alcohol, fruit and ‘unhealthy’ food consumption, eating foods they believed had healing properties, engagement in foot self care and increased physical activity.

I was never buying anything with more than 8% sugar in. Eating lots of yellow fruits: bananas, grapefruits, lemons… (PWD21_Male_60–75 years)

I think they acknowledge that they are almost like the masters of their own destiny sometimes and want to know what they can do. (HCP3_Doctor_5–7 years' experience)

People with T2DM expressed that the positive dietary behaviours they engaged in as a result of their DFU were driven by prior knowledge or information‐seeking, not from HCP advice, since dietary information seldom featured in foot‐focused appointments:

Very little … it is published in books but I don't think it's—it's not made very aware … one has to do their own research on that. (PWD3_Male_60–75 years)

HCPs recognised diet as an area where knowledge was lacking, and given that the appointments were foot‐focused, diet was not a priority. Access to dietary support was also scarce, with referral waiting times up to one year.

I'd probably say we don't talk about diets … of all of the things that we talk about—smoking, blood pressure—diet is a thing that probably comes right at the bottom … it's probably the thing we don't have time to discuss or the thing as podiatrists, we don't have the skills to discuss. (HCP2_Podiatrist_9+ years' experience)

##### 3.1.2.2 Fostering unhealthful behaviours

DFUs could foster behaviours that negatively impacted health. This included being sedentary due to HCP advice to engage in minimal activity during DFU healing and because being mobile risked further ulcer development.

I was told I got to keep off my foot … keep as much weight off it as possible and not walk on it. (PWD4_Male_40–59 years)

I've got a patient who doesn't get off the sofa because he said “I'm fine, I don't re‐ulcerate”, because he's had amputations and things, he said “I don't re‐ulcerate as long as I just sit here”…. (HCP21_Podiatrist_9+ years' experience)

Incentives to make positive physical activity changes could be curtailed due to the perception held by HCPs and individuals with T2DM that DFU healing requires sedentary behaviour. Those holding this belief viewed exercise as encompassing weight‐bearing or lower‐body activities only, they did not consider upper‐body alternatives:

I couldn't do anything because basically, to do any kind of exercises you need to stand up. (PWD4_Male_40–59 years)

One of the problems, obviously, with a DFU … they can't exercise once they've got an ulcer. (HCP14_Podiatrist_9+ years' experience)

Adults with T2DM discussed how, with active DFUs, they were unable to stand in the kitchen and cook, resulting in a reliance on processed foods, or food made by others where they had no control over the nutritional content:

I've been doing a lot of ready meals. (PWD15_Female_60–75 years)

The most commonly discussed unhealthful change in dietary behaviour(s) was increased food consumption (quantity or frequency) or ‘comfort eating’ (quality), due to boredom, anxiety or changes in mental health:

Whilst I've been enforced sedentary, I probably eat more as a result of either being bored or anxious about recovery. (PWD18_Male_60–75 years)

##### 3.1.2.3 Behaviours discordant with recommended advice

Optimal DFU healing often required people to change their behaviours. However, adults with T2DM described not always following DFU treatment recommendations, resulting in behaviours discordant with HCPs' advice. Not adhering to advice was driven by several factors, including not wearing prescribed footwear due to the perceived social implications, struggles with mental health meaning the ‘desired’ behaviours were difficult to engage with, and a lack of support or motivation to change:

It [prescribed footwear] was extremely awkward and uncomfortable, and I'll be perfectly honest … I work at an office where I'm a senior manager here, and I never used to wear it. (PWD14_Male_40–59 years)

I do sometimes think that there are many issues with our diabetic foot patients around giving advice, because they're not always very receptive to the advice we may give them, and I think it's around the fact that once you've had a problem and complications of a disease, you haven't got the motivation to then change things. (HCP9_Podiatrist_9+ years' experience)

Both cohorts recognised DFUs can remove someone's autonomy, making them more dependent on others and unable to engage in activities they enjoy. This meant people with T2DM might hold on to what they can do or feel they can control, which may not align with treatment advice:

I … walk round the park … and say sod the foot, but I know it's going to set it back’. (PWD9_Male_60–75 years)

A lot of patients say, to me, “it's [smoking] the only pleasure I've got” … stopping smoking is one very difficult thing to encourage … when there's so much negativity in their lives. (HCP19_Podiatrist_9+ years' experience)

Discordant behaviours were likely to occur when treatment recommendations were not sensitive to individual needs. HCPs' understanding of someone's employment situation was imperative to encourage appropriate treatment plans, as some individuals cannot take time off work or wear prescribed footwear without risking their income. HCPs advised that there are new challenges as the incidence of T2DM rises among younger people, who they perceive as having more to navigate in their lives. If HCPs' recommendations are not aligned with each individuals' needs and circumstances, caring for DFUs may be prioritised less by younger adults:

It's more difficult really for them [younger people], and for the provision of care, because many of them are working and they don't always have access to funds … So, they are expected to return to work … Obviously, that will impact on the amount of compliance or co‐production that we can have with their treatment, as in offloading them. They're not always allowed to wear things that we will provide in their workplace. (HCP17_Podiatrist_9+ years' experience)

#### 3.1.3 Physical impact of DFUs

DFUs immobilised individuals for long periods of time, in some cases up to several months if ulcers do not heal or complications occurred. Both cohorts advised that sustained inactivity could lead to immobility and loss of strength, which could impact a person's ability to be active even once DFUs had healed. Pain arising from ulcers or off‐loading devices could further impact mobility:

The first time I was non‐weight bearing, when I got up to walk, my legs were so weak that I couldn't do it. (PWD22_Female_40–59 years)

Prolonged inactivity could also lead to increases in weight and, as HCPs advised, an increased risk of further diabetes‐related complications, including high blood pressure and CVD. It was evident that the physical manifestations of DFUs can influence one another. For example, inactivity and reduced mobility can lead to further complications and increased weight, and increased weight can lead to further complications and reduced mobility:

It's tricky because when someone has a DFU, we are generally asking them to offload their foot…And that is often associated with their diabetes becoming a little bit worse controlled, their weight going up, all of those things that in turn heighten their CVD risk. (HCP2_Podiatrist_9+ years' experience)

#### 3.1.4 Psychosocial impact of DFUs

DFUs could negatively affect mental health and quality of life, and foster worry and concern about the future; whether individuals would be mobile again to resume day‐to‐day and social activities, and about the possibility of recurrent or more complex DFUs.

It's obviously had some effect on my mental health over that time … It's really hampered my social life and the ability to just walk the dog half the time. (PWD9_Male_60–75 years)

Loss of mobility equals loss of independence and that can have massive knock‐on effects with mental health…. (HCP16_Nurse_1–3 years' experience)

Several individuals with active DFUs had accepted, or were in the process of accepting, the impact of DFUs, including the restrictions they placed on their lives and the loss of their previous self. Acceptance meant relinquishing their life before and appeared to be on a continuum, with some individuals having reached it, and some still coming to terms with what they had lost. Time, older age and social support seemed to help foster acceptance:

It's slowly dawning on me I'm not going to be like I was three years ago … I can't see that happening. So, there's an element of me is coming to terms with what I've lost. (PWD7_Male_60–75 years)

### 3.2 Unmet needs of people with T2DM and DFUs

Several areas of unmet need were identified from the interviews: treatment plans devised through shared decision making, and access to appropriate physical well‐being and psychological support services.

#### 3.2.1 Shared‐decision making to aid the development of appropriate treatment plans

Participants with DFU highlighted the necessity and current paucity of HCPs understanding the context of someone's life in order to advise on appropriate treatment options. Both cohorts felt HCPs should agree on treatment plans with individuals, rather than HCPs dictating what to do, which may not be feasible. Time, continuity of care, empathy and listening skills facilitated this process. Inappropriate advice risked fracturing relationships with HCPs, which could further encourage behaviours detrimental to DFUs:

Listening, empathy, trying to understand … not being too judgemental … try and understand their social position, you know, what's happening for them in the rest of their lives and work with them. (HCP21_Podiatrist_9 years+ experience)

I got to see her and I built up the trust and we stopped her walking. We built her activity up slowly. We made different footwear adaptions. (HCP6_Podiatrist_9+ years' experience)

#### 3.2.2 Psychological support

In response to the psychosocial impact of DFUs, participants expressed how treatment often centred on medication:

The treatment I'm getting for ulcers, nine times out of ten, do have an effect on my mental health, but there's no mental health support … The only support I have are antidepressant tablets. (PWD15_Female_60–75 years)

HCPs felt that access to psychological support could help individuals cope with the impact of DFUs. However, the availability of support services and their accessibility was dependent on locality:

There used to be a psychologist that was aligned with the community diabetes team that we could refer people to if they were struggling with making lifestyle changes or adapting to the new normal … But that, as with lots of NHS posts, that post is currently vacant. So, we don't have that level of expertise. (HCP10_Podiatrist_7–9 years' experience)

In circumstances where streamlined referrals to psychological support were in place, or psychologists were part of the multidisciplinary team providing foot care, individuals were provided with specialist care to help them adapt to life with DFUs. This not only benefitted individuals, but HCPs as well:

For patients … you believe are struggling psychologically … we will refer [to IAPT: Improving Access to Psychological Therapy], and it works quite well. We then sit together as a part of an MDT [multi‐disciplinary team] where certain behavioural changes or challenges can be discussed, and the MDT team can think of ways of manoeuvring or navigating around it. (HCP12_Podiatrist_7–9 years' experience)

#### 3.2.3 Physical well‐being support

Increased support was needed in response to the physical impacts of DFUs. Participants discussed delays in referrals for physical support coming to fruition, and even when they did, the care did not always fulfil an individual's needs; some participants with DFUs described it as minimal, unsuitable and hands‐off. HCPs further advised that physiotherapy support was only available to specific cohorts, for example those experiencing multiple problems or pain. In light of this, some individuals with DFU were not receiving access to, or adequate, physical support despite a demonstrable need:

He [physiotherapist] was pretty poor…came once, nice guy, but actually didn't do much. He gave me a set of crutches which were the wrong size and then he told me he was discharging me because he's moving jobs. (PWD7_Male_60–75 years)

When individuals with DFUs received specialist physical activity support that matched their needs, they described significant improvements:

They [physiotherapists] talked about the exercises on the floor or the bed, and then obviously when I got my walker, it was walking different distances in the house … walking through to the kitchen and turning around safely … walking out in the street. Walking so far, resting, and that sort of thing … they were so good in bringing me on, I was looking for some more. (PWD13_Male_75+ years)

Whilst exercise or behavioural programmes were available in some localities to support people, these were not always suitable for the group since they focused on weight‐bearing activities. Further, not all HCPs felt knowledgeable enough to provide non‐weight bearing activity advice to assist with the physical impacts of DFUs and would want to be upskilled or have exercise expertise available to the team:

When you look at the physiotherapy team … they will focus on rehabilitation of post‐surgery or stroke, neurological conditions. There's no experts, physiotherapists in diabetes, you know, foot disease, which I think is really, again, a real big missed opportunity. That's something that we should be challenging. (HCP6_Podiatrist_9+ years' experience)

HCPs who had been upskilled or had strong links with physiotherapy colleagues felt more competent in prescribing exercises, demonstrating the merit of close working relationships with exercise specialists for the physical well‐being of people with DFUs:

We're already aware of quite a few exercises one can prescribe … So, we collaborate quite closely with our physio or rehab colleagues. (HCP12_Podiatrist_7–9 years' experience)

## 4 DISCUSSION

This study explored the experiences and unmet needs of people living with T2DM and DFUs through interviews with those living with these conditions and HCPs involved in their care.

Key findings included a lack of awareness of foot ulcers and their association with T2DM, echoing findings from previous UK studies.^18^, ^19^ This lack of awareness persists despite earlier research calling for reform of DFU education. However, in light of the barriers highlighted in our study and previous research (i.e. low health literacy), and the literature indicating DFU knowledge alone does not change behaviour,^20^ it is not only about providing education, but ensuring that individuals understand and are engaged with the information and behaviour change. Attention needs to be directed towards factors affecting understanding of and responses to DFU information,^21^ looking mutually at the role of the individual with T2DM and HCPs. This can help avoid people getting to the point of DFUs and experiencing shock and self‐blame, or placing blame on HCPs and the health service.

Our findings show that experiencing DFUs prompted positive lifestyle changes. Participants in our study were motivated to change their dietary behaviours, however as highlighted in previous research, people with DFU report not receiving any dietary advice during foot‐focused consultations or being signposted to nutrition services.^22^ This meant any changes made were often self‐directed, which may lead to diet misconceptions or unsubstantiated dietary changes if incorrect advice is obtained, for example that fruit consumption impedes ulcer healing.^22^ In contrast to the positive changes discussed, DFUs could also negatively impact dietary behaviours. As previously reported, some individuals relied on processed foods,^22^ and our findings suggested ‘comfort eating’ was a further by‐product of DFUs. This could lead to increased weight, which can increase a person's risk of additional health complications, including CVD.^23^ Given that those living with DFUs are already vulnerable to accelerated cardiovascular events and mortality, and diet is key for helping manage CVD risk, more is needed to support people with DFUs to maintain healthy diets and engage in effective behaviour change. Echoing previous research, boredom and negative feelings^24^ were triggers for ‘comfort eating’. HCPs opening up dialogue about how DFUs may be affecting someone's well‐being and behaviours may help identify if the condition is affecting diet, with signposting to resources or specialist referrals offered where appropriate. Dietary support is important since malnutrition and glycaemic levels can impact DFU healing.^25^

Individuals with DFUs and HCPs often reported a shared view that those with active ulcers need to engage in minimal activity, without considering upper‐body alternatives. Beliefs around being active with DFUs need to be challenged to encourage non‐weight‐bearing forms of activity for the benefit of individuals' cardiovascular health. However, many HCPs did not feel knowledgeable enough to prescribe exercise, especially given that the population often presents with frailty and co‐morbidities.^26^ Prescribing upper‐body exercise could particularly benefit those who report feeling anxious and bored when offloading DFUs. A recent study^27^ reported that individuals found difficulty using off‐loading devices as recommended due to finding enjoyment through exercise, yet it may be possible to achieve such enjoyment through upper‐body exercises. Our findings further suggest that inactivity due to DFUs can lead to low mood and depressive symptoms^13^; activity through non‐weight‐bearing exercise could help counteract this.

A need for improved access to specialist physical activity support (e.g. physiotherapists) and tailored exercise programmes for the DFU population was highlighted to better support their physical well‐being. Regular exercise reduces the risk of CVD and early mortality,^28^ and has also been found to improve symptoms of depression and anxiety.^29^ This is noteworthy given the CVD risk of the population, and that half of individuals with DFU report experiencing depressive symptoms.^8^ The physical needs of the DFU population (e.g. being non‐weight bearing, recovering from amputation) necessitates adapting exercises to each individual's needs. A systematic review found that tailoring physical activity for older adults in the community reduced the symptoms of diseases, including CVD, alongside improving physical outcomes (e.g. mobility, balance, muscle strength).^30^ However, no positive impact on exercise adherence was found. If considering tailoring exercise interventions to the needs of people with DFU, those with lived experience should be involved to promote suitability.^S1^ Our research also found that access to physiotherapy or exercise specialists was often delayed or limited, leaving individuals with DFU in need. Further, exercise, lifestyle and weight loss programmes frequently focused on weight‐bearing activity, making them unsuitable for people who cannot bear weight, further supporting the need to develop bespoke interventions for the DFU population.

It was evident across both cohorts that how well a person's ulcer heals is often determined by their behaviour, and a person's ability to change their behaviour can be affected by psychological, social and environmental factors. As suggested by earlier research, having processes in place to elicit information about the context of people's lives can help individuals and HCPs engage in shared decision making to mutually agree upon suitable DFU care plans.^S2,S3^ Although shared decision making between people with DFU and HCPs was not found to improve engagement with foot treatments in an earlier study,^S4^ as reported by the authors, a lack of motivation could have been a barrier to making changes despite implementing co‐designed treatment plans. In line with this, our participants commonly reported a lack of motivation to change stemming from struggles with mental health brought on by living with DFUs. However, access to non‐pharmacological psychological support (e.g. talking therapies) was scarce, despite recognition that these therapies are imperative for those in need to support psychological well‐being, and to help remedy the lack of motivation experienced by people with DFUs. Being psychologically well helps lay the foundation for positive behaviour changes and their maintenance.^13^

Overall, our findings underscore the importance of a holistic, multidisciplinary approach to providing care for people with DFUs (e.g. incorporating dietary, physical activity and psychological support), guided by person‐centred care and shared decision making.^S3^

In our study, HCPs discussed how younger adults with T2DM are developing DFUs more frequently. Evidence shows that the risk of DFU increases with age,^S5^ with recent research indicating that individuals with T2DM who develop a DFU are on average 64.5 years of age.^S6^ This suggests the need for a better understanding of how to integrate DFU care into the lives of younger adults compared to older individuals,^S6^ especially given previous research indicates that younger age is associated with longer DFU healing times^S7^ and ulcer recurrence.^S8^ Future research should seek to elucidate young adults' views of DFUs to identify their specific healthcare needs, in addition to preventing the occurrence of DFUs in the first instance within the younger age range (e.g. 18–45 years). This is a crucial area of research given the rapid increase in younger adults being diagnosed with T2DM.^S9^

## 5 STRENGTHS/LIMITATIONS

This qualitative study aimed to inform holistic care pathways for individuals living with T2DM and DFUs through exploring their experiences and unmet needs. A key strength is that we interviewed two cohorts to capture different views and experiences. Given that people with DFU and HCPs need to work together upon the onset of an ulcer to help promote optimal health and well‐being, we feel it is important to understand both groups' beliefs, opinions and experiences. Further, the TDF was used to underpin the topic guides, which provides a systematic and evidence‐based approach to qualitative data collection.^16^ The dearth of variation in age, gender and ethnicity of the T2DM and DFU participants, and profession in HCPs, meant a limited number of perspectives were explored. However, the characteristics of the participants with DFU reflect the DFU population within the United Kingdom which is predominantly male, older in age and of White ethnicity.^S10^

## 6 CONCLUSION

In response to the behavioural, psychosocial and physical impacts of DFUs, and the lack of knowledge and awareness of the association between T2DM and DFUs, this study has highlighted several important areas of unmet need among adults with T2DM and DFUs. These include improved, personalised and sustained access to: DFU education, behaviour change support through shared decision making, with input from psychological support services, and tailored physical activity interventions and specialist services. This can be achieved through a multidisciplinary approach to providing care to the population.

## AUTHOR CONTRIBUTIONS

MH oversaw the qualitative study. MC conducted the interviews, carried out data analysis and drafted the manuscript with support from MH. All qualitative study documentation was developed by MH, AG and MC. JS contributed to the review of data analysis and interpretation. AG, MC, MH and KK supported and aided participant recruitment. AG, PH, JS and KK read and agreed on the final manuscript.

## FUNDING INFORMATION

This study forms part of the MiFoot research programme: Developing and Evaluating A Multifactorial Intervention to Improve Cardiovascular Outcomes in Adults with Type 2 Diabetes and Current or Previous Diabetic Foot Ulcers (MiFoot). MiFoot is funded by the National Institute for Health and Care Research (NIHR) under its Programme Grants for Applied Research Programme (NIHR202021). The views expressed are those of the author(s) and not necessarily those of the NIHR or the Department of Health and Social Care. JS is supported by the funding provided to the ACBRD as part of the collaboration agreement between Diabetes Victoria and Deakin University.

## CONFLICT OF INTEREST STATEMENT

KK is supported by the National Institute for Health Research (NIHR) Applied Research Collaboration East Midlands (ARC EM), NIHR Global Research Centre for Multiple Long Term Conditions, NIHR Cross NIHR Collaboration for Multiple Long Term Conditions, NIHR Leicester Biomedical Research Centre (BRC) and the British Heart Foundation (BHF) Centre of Excellence. All other authors declare no conflicts of interest.

## ETHICS STATEMENT

Research Ethics Committee (REC) favourable opinion and HRA approval were obtained from the London—Brent Research Ethics Committee (22/PR/0272). All participants gave informed consent prior to their participation in the study.

## Supporting information


Data S1.

## Data Availability

The data supporting the findings of this study are not publicly available, and the availability of the data is subject to restrictions used under license for the current study. However, data can be made available from the authors upon request and with permission of the Leicester Diabetes Centre.
