# Supplementary material for: A qualitative study exploring and understanding the experiences and unmet needs of adults with type 2 diabetes and diabetes‐related foot ulcers
Source: Diabet Med. 2026 May 4;43(11):e70343. doi: 10.1111/dme.70343 (PMC13669981; doi:10.1111/dme.70343)
Supplement: Supplementary file 1 [file DME-43-e70343-s001.docx]

S1.	Ma J, Floegel T, Li L, et al. Tailored physical activity behavior change interventions: challenges and opportunities. *Translational Behavioral Medicine*. 2021;11(12):2174-2181. doi.org/10.1093/tbm/ibab106

S2.	Van Netten JJ, Seng L, Lazzarini PA, Warnock J, Ploderer B. Reasons for (non‐)adherence to self‐care in people with a diabetic foot ulcer. *Wound Repair and Regeneration*. 2019;27(5):530-539. doi:10.1111/wrr.12728

S3.	Zamani N, Chung J, Evans-Hudnall G, et al. Engaging patients and caregivers to establish priorities for the management of diabetic foot ulcers. *Journal of Vascular Surgery*. 2021;73(4):1388-1395. doi:10.1016/j.jvs.2020.08.127

S4.	McBride E, Hacking B, O'Carroll R, et al. Increasing patient involvement in the diabetic foot pathway: a pilot randomized controlled trial. *Diabetic Medicine*. 2016;33(11):1483-1492. doi:10.1111/dme.13158

S5.	McDermott K, Fang M, Boulton AJM, Selvin E, Hicks CW. Etiology, Epidemiology, and Disparities in the Burden of Diabetic Foot Ulcers. *Diabetes Care*. Jan 1 2023;46(1):209-221. doi:10.2337/dci22-0043

S6.	Røikjer J, Werkman NCC, Ejskjaer N, et al. Incidence, hospitalization and mortality and their changes over time in people with a first ever diabetic foot ulcer. *Diabetic Medicine*. 2022;39(4). doi:10.1111/dme.14725

S7.	Zhang Y, Cramb S, McPhail SM, et al. Factors Associated With Healing of Diabetes-Related Foot Ulcers: Observations From a Large Prospective Real-World Cohort. *Diabetes Care*. 2021;44(7):e143-e145. doi:10.2337/dc20-3120

S8.	Hicks CW, Canner JK, Mathioudakis N, Lippincott C, Sherman RL, Abularrage CJ. Incidence and Risk Factors Associated With Ulcer Recurrence Among Patients With Diabetic Foot Ulcers Treated in a Multidisciplinary Setting. *Journal of Surgical Research*. 2020/02/01/ 2020;246:243-250.

S9.	Nanayakkara N, Curtis AJ, Heritier S, et al. Impact of age at type 2 diabetes mellitus diagnosis on mortality and vascular complications: systematic review and meta-analyses. *Diabetologia*. 2021;64(2):275-287. doi:10.1007/s00125-020-05319-w

S10.	Riley J, Antza C, Kempegowda P, et al. Social Deprivation and Incident Diabetes-Related Foot Disease in Patients With Type 2 Diabetes: A Population-Based Cohort Study. *Diabetes Care*. 2021;44(3):731-739. doi:10.2337/dc20-1027
